# CARD11 regulates the thymic Treg development in an NF-κB-independent manner

**DOI:** 10.3389/fimmu.2024.1364957

**Published:** 2024-04-08

**Authors:** Yu Hu, Lingli Han, Wenwen Xu, Tianci Li, Qifan Zhao, Wei Lu, Jinqiao Sun, Ying Wang

**Affiliations:** ^1^ Chinese Academy of Sciences (CAS) Key Laboratory of Tissue Microenvironment and Tumor, Shanghai Institute of Nutrition and Health, University of Chinese Academy of Sciences, Chinese Academy of Sciences, Shanghai, China; ^2^ Department of Clinical Immunology, Children’s Hospital of Fudan University, National Children’s Medical Center, Shanghai, China; ^3^ Key Laboratory of Neonatal Diseases, Ministry of Health, Children’s Hospital of Fudan University, Shanghai, China

**Keywords:** CARD11, immunodeficiency, regulatory T cells, development, FOXO1

## Abstract

**Introduction:**

CARD11 is a lymphoid lineage-specific scaffold protein regulating the NF-κB activation downstream of the antigen receptor signal pathway. Defective CARD11 function results in abnormal development and differentiation of lymphocytes, especially thymic regulatory T cells (Treg).

**Method:**

In this study, we used patients’ samples together with transgenic mouse models carrying pathogenic *CARD11* mutations from patients to explore their effects on Treg development. Immunoblotting and a GFP receptor assay were used to evaluate the activation effect of CARD11 mutants on NF-κB signaling. Then the suppressive function of Tregs carrying distinct CARD11 mutations was measured by *in vitro* suppression assay. Finally, we applied the retroviral transduced bone marrow chimeras to rescue the Treg development in an NF-κB independent manner.

**Results and discuss:**

We found CARD11 mutations causing hyper-activated NF-κB signals also gave rise to compromised Treg development in the thymus, similar to the phenotype in Card11 deficient mice. This observation challenges the previous view that CARD11 regulates Treg lineage dependent on the NF-kB activation. Mechanistic investigations reveal that the noncanonical function CARD11, which negatively regulates the AKT/ FOXO1 signal pathway, is responsible for regulating Treg generation. Moreover, primary immunodeficiency patients carrying CARD11 mutation, which autonomously activates NF-κB, also represented the reduced Treg population in their peripheral blood. Our results propose a new regulatory function of CARD11 and illuminate an NF-κB independent pathway for thymic Treg lineage commitment.

## Introduction

As an essential part of central tolerance, the regulatory T cell (Treg) is a professional cell type that maintains immune homeostasis by suppressing autoimmune responses (1, 2). There are multiple developmental origins for Tregs. Tregs naturally generated *in vivo* are called nTreg and consist of two sub-types: tTreg, committed in the thymus, and pTreg, which differentiates from naive CD4^+^ T cells in the peripheral tissues or organs. *In vitro*, specific stimuli, combining T cell receptor (TCR) signaling and cytokine supplementation, induce naive CD4^+^ T cell differentiation into iTreg (3, 4). Like many other hematopoietic origin cell types, tTregs initiate their development trajectory in the bone marrow. Bone marrow-derived T cell precursors migrate to the thymus and then develop into CD4^-^ CD8^-^ double-negative cells, followed by CD4^+^ CD8^+^ double-positive cells, on which the rearranged TCRs are functionally assembled (5). Then, TCR signaling facilitates the positive and negative selection of thymocytes, which enforces the central tolerance in the thymus. Most T cell clones with TCRs that recognize self-antigens are eliminated. However, some CD4^+^ single positive (SP) T cells with moderate responsiveness against an autoantigen are not clonally deleted; instead, they further differentiate into tTreg cells (5, 6). Therefore, TCR signaling is critical for tTreg lineage determination.

Molecular regulations of tTreg commitment are complicated, and many critical epigenetic and transcriptional factors have been discovered (7). For example, the autoreactive CD4^+^ SP T cells in the thymus produce a substantial amount of IL-2, activating the downstream transcription factors (TF) STAT5 in tTreg precursors. STAT5 promotes the expression of *FoxP3*, the dominant TF of the Treg lineage, and many other Treg-specific genes (8–10). Besides STAT5, Smad2 downstream of TGF-β signaling also facilitates the tTreg development in the thymus (11). As the most critical cell surface receptor on Tregs, TCR engagement also activates various downstream TFs, including NF-κB, to establish the tTreg-specific transcriptome. Lack of NF-κB components, p65 or c-Rel, severely disrupted the tTreg development in mice (12).

Many signaling proteins cooperate delicately to transduce antigen stimulation signals from the cell surface to activate the downstream NF-κB. CARD11 is a scaffold protein indispensable for such signal transductions for the tTreg development (13, 14). After antigen stimulation, CARD11 is phosphorylated by the protein kinase PKCθ downstream of the TCR (15). The stimulation-induced phosphorylation changes the conformation of CARD11 from an auto-inhibition form into an active form, permitting the association of the Bcl10-MALT1 heterodimer and constructing a signal transduction hub called the CBM complex. Finally, the CBM complex triggers the TRAF6-TAK1-NEMO-IKKβ signal cascade, igniting NF-κB-related transcription (16). *Card11* deficiency dramatically compromises the NF-κB signal pathway after TCR stimulation, disrupting tTreg development but leaving other T cell development uninfluenced (17, 18). Both hypo- and hyper-activated CARD11 have been associated with various immunological disorders, and the pathological defects result from the dysregulation of NF-κB. For example, somatic gain-of-function (GOF) CARD11 mutations were found in nearly 10% of the activated b-cell type, diffused-large b-cell lymphoma (ABC-DLBCL), which depends on elevated NF-κB activity for survival (19, 20). The loss-of-function (LOF) mutations of CARD11 were associated with severe primary immune deficiency (PID), and patients without CARD11 suffered from reoccurring infections and other symptoms caused by compromised immune systems (21). Hypomorphic CARD11 mutations also cause severe atopic dermatitis with or without comorbid infections (22), and this disease was named CARD11-associated atopy with dominant interference of NF-κB signaling (CADINS) disease (23). Interestingly, the germline GOF mutation of CARD11 also leads to a PID called BENTA (B cell Expansion with NF-κB and T cell Anergy) disease (24), representing mainly a B cell phenotype but also T cell abnormalities.

The detailed mechanisms underlying pathogenic CARD11 mutants remain unclear. Particularly, will the GOF CARD11 mutant impact Treg development and contribute to the pathogenesis? Therefore, we applied transgenic mouse models with distinct CARD11 mutations and explored the tTreg development in the CARD11 mutant genetic backgrounds.

## Materials and methods

### Mice

*Card11* E134G, K215M mutant, and *Card11* KO C57BL/6 mice were generated as described previously (25). All mice were maintained and bred in a specific pathogen-free facility. Mouse experimental protocols were approved by the Institutional Biomedical Research Ethics Committee of the Shanghai Institute of Nutrition and Health Science, Chinese Academy of Sciences.

### Human samples

The Ethics Board of Children’s Hospital, Fudan University, approved all the patient-related studies. Whole blood samples were obtained from patients and healthy controls using vacutainer tubes containing EDTA anticoagulant. Peripheral blood mononuclear cells (PBMC) were isolated by density centrifugation with Human Lymphocyte Separation Medium (Dakewe Biotech), followed by red blood cell lysis buffer (Biolegend) treatment. Isolated PBMCs were tested by flow cytometric analysis or cryopreserved in media containing 90% heat-inactivated fetal bovine serum (FBS) and 10% DMSO.

### Cell preparation from mice and flow cytometry

Mouse spleen and thymus were collected and mashed through a 40-µm cell strainer to generate single-cell suspensions, followed by red blood cell lysis. Cells were then blocked with Fc receptor antibody (Cat. BE0307, Bio X cell) for 15 minutes at 4°C before staining with fluorochrome-conjugated surface antibodies: Viability Dye (Cat. 65-0865-14), CD45.1 (clone: A20, BioLegend), CD45.2 (clone: 104, BioLegend), CD4 (clone: GK1.5, Biolegend), CD8 (clone: 53-6.7, BioLegend), CD25 (clone: PC61, BD), CD69 (clone: H1.2F3, BD). After surface staining, cells were fixed with fixation/permeabilization buffer (Cat.00-5123-43, eBioscience) and then permeabilized and stained in permeabilization buffer (Cat. 00-8333-56, eBioscience). Antibodies for intracellular staining are FoxP3 (clone: FJK-16s). Flow cytometric analysis was performed using a Gallios flow cytometer. Data were processed and analyzed through FlowJo software.

### Western blot

Equal amounts of protein from each sample were denatured in the loading buffer by heating for 20 minutes at 100°C. Samples were separated by sodium dodecyl sulfate-polyacrylamide gel electrophoresis (SDS-PAGE) on 8%-10% polyacrylamide bio-tris gels and transferred to nitrocellulose membranes. After transfer, nitrocellulose membranes were blocked in 5% milk for up to 1 hour at room temperature, followed by incubation with primary antibodies against phosphor-AKT (T308), phosphor-AKT (S473), phosphor-FOXO1, total FOXO1, total AKT, and mouse anti-actin (Cell Signaling Technology) at 4°C overnight. Membranes were washed with PBST and then incubated with HRP-conjugated secondary antibodies. Proteins were visualized and quantitated by the Bio-Rad ChemiDoc XBS System.

### *In vitro* activation and differentiation of naïve CD4^+^ T cells

Naïve CD4^+^ T cells were purified using the MojoSort™ Mouse Naïve CD4^+^ T cell isolation kit (Cat. 480033, Biolegend) following the manufacturer’s protocol. Naïve CD4^+^ T cells were then activated and differentiated by plating at a density of 1x10^6^ cells per well on coated anti-CD3 (2µg/mL, Cat.BE0001-1, BioXcell) and anti-CD28 (2µg/mL, Cat. BE0015-1, BioXcell) 48-well plates for four days in 37 °C incubators with 5% CO_2_ with IL-2. IL-12 and anti-IL-4 were added to growth media to promote Th1 differentiation. IL-4, anti-IFN-γ, and anti-IL-12 were added to growth media to promote Th2 differentiation. TGFβ, IL-6, IL-1β, IL-23, anti-IL-4, anti-IL-12, and anti-IFN-γ were added to growth media to promote Th17 differentiation. TGFβ, anti-IL-4, anti-IL-12, and anti-IFN-γ were added to growth media to promote iTreg differentiation. After differentiation, cells were collected, washed with PBS, and analyzed by flow cytometry.

### Human T cell transfection

Isolated PBMCs were transfected with WT and mutated *CARD11* plasmids, respectively, by using the Human T Cell Nucleofector™ Kit (Cat.VPA-1002, Lonza). Phosphor-AKT (T308) in CD4^+^ T cells was detected by flow cytometry after 24 hours.

### NF-κB induced GFP reporter assay

HEK-293T cells were transfected with NF-κB reporter plasmids together with EV (Empty Vector), WT *CARD11*, and mutation plasmids, respectively, using polypropyleneimine (PEI). 24 hours after transfection, NF-κB induced GFP fluorescence in transfected HEK-293T cells was detected by flow cytometry.

### Treg suppression assay

CD25^high^ Tregs from CD45.2^+^ WT, heterozygous E134G, and homozygous K215M mutant mice were purified by flow cytometry sorting. Total CD4^+^ T cells of CD45.1^+^ WT mice were purified with the MojoSort™ Mouse CD4^+^ T cell isolation kit and stained with CFSE. CD45.1^+^ and CD45.2^+^ antigen-presenting cells were treated with mitomycin (20µmol/mL). Then, all the cells above were cocultured with anti-CD3 (1µg/mL) and IL-2 (10ng/mL) for 4 days. CFSE fluorescence intensity was analyzed by flow cytometry.

### Retroviral transduced bone marrow chimera mouse model

Mouse *Foxo1* cDNA was cloned into an MSCV-IRES2-EGFP retroviral vector. Retrovirus was made by transfection of HEK-293T cells. BM cells were collected from the femurs and tibiae of 6- to 8-week-old donor mice. After red blood cell lysis, hematopoietic stem cells were enriched using the Lineage Cell Depletion Kit (Miltenyi Biotec). Hematopoietic stem cells were cultured in chemically defined serum-free medium X-VIVO 10 with gentamicin (Lonza) supplemented with L-glutamine, β-mercaptoethanol (50 mM), mouse recombinant stem cell factor (50 ng/mL), IL-6 (20 ng/mL), IL-3 (10 ng/mL), FLT-3L (10 ng/mL), and IL-7 (10 ng/mL) (PeproTech) for 24 hours. Then, cocultured cells were transduced by spin infection with retroviral supernatant. Cells were incubated for another 24 hours before intravenous tail injection into WT recipient mice, which were irradiated at 9 Gy for at least 12 hours before adoptive transfer. 6 weeks after transfer, chimeras were analyzed by flow cytometry.

### Statistical analysis

Two-tailed *t*-tests were used for two-data group comparisons, and multiple *t*-test statistical significance used the Holm-Sidak method for multiple comparisons. Statistical tests were run using GraphPad software. SEM was reported for all experiments, and P<0.05 was considered statistically significant.

## Results

### Pathogenic CARD11 mutants have distinct impacts on tTreg development

**Figure 1:**
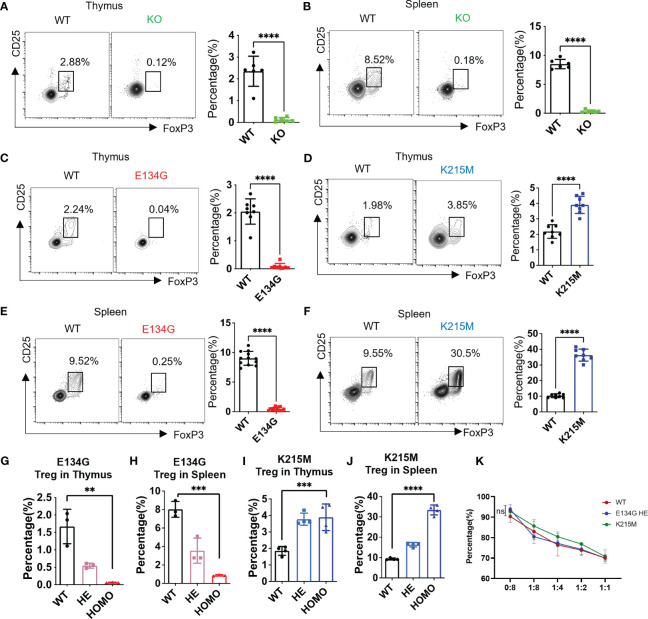
Pathogenic CARD11 mutants have distinct impacts on tTreg development. **(A, B)** CD25 and FoxP3 expression on CD4^+^ T cells in the thymus and **(B)** spleen of WT and *Card11* KO mice were analyzed by flow cytometry (left). The quantifications of the CD25^+^ FoxP3^+^ tTreg cells in WT and *Card11* KO mice (n=6) (right). **(C)** CD25 and FoxP3 expression on CD4^+^ T cells in the thymus of WT and *Card11* E134G mice, as well as of WT and *Card11* K215M mice **(D)**, were evaluated by flow cytometry (left). The CD25^+^ FoxP3^+^ tTreg population frequency in WT and *Card11* mutant mice (n=8) was shown in the right panel. **(E, F)** Flow cytometry analysis of CD25 and FoxP3 expression on CD4^+^ T cells of the spleen from WT and *Card11* E134G mice as well as **(F)** WT and *Card11* K215M mice (left). The frequency of the Treg population in WT and *Card11* mutant mice (n=8) was shown in the right panel. **(G)** The frequency of Treg cells in the thymus and **(H)** spleen of WT, heterozygous, and homozygous E134G mutant mice (n=3). **(I)** The frequency of Treg cells in the thymus and **(J)** spleen of WT, heterozygous, and homozygous K215M mutant mice (n=4). **(K)** Tregs purified from WT, heterozygous E134G, and homozygous K215M mutant mice were cocultured with WT CD4^+^ T cells for 4 days (stimulated with antigen-presenting cells, 1μg/mL anti-CD3 antibody, and 10ng/mL IL-2). The CD4^+^ T cell proliferation in three groups was measured by flow cytometry, and the data was analyzed with multiple *t-*tests (n=3). Data represent the mean ± SEM of n>3 biological replicates. **P<0.01, ***P<0.001, ****P<0.0001; P values were determined using unpaired two-tailed Student’s *t-*tests.

To better understand the pathogenic role of CARD11 mutations, we established various *Card11* transgenic mouse models with corresponding genetic modifications (25). The E134G mutant mice were used to investigate the pathogenesis of BENTA disease (24). And the K215M mutant mimics the DLBCL (19). Finally, the *Card11* KO mice represent the PID patients without CARD11 (**Supplementary Figure 1A**). We compared the morphology of the thymus and spleens of WT, E134G, and K215M mutant mice (**Supplementary Figures 1B, C**). There was no significant difference in thymus size. However, the spleen of the K215M mutant mice is larger than that of the WT and E134G mutant mice. That may be due to the aberrant B cell activation and proliferation in K215M mutant mice, as reported before (25). Then, we checked the Treg development in the thymus of those distinct *Card11* transgenic mouse models. In *Card11* KO thymus, the tTreg population was significantly decreased, as in the previous report (**Figure 1A**). This dramatic Treg reduction also led to less mature Tregs in the KO spleen (**Figure 1B**). We then explored the Treg development under the CARD11 mutant background. The E134G mutant was identified in BENTA disease patients and autonomously induced NF-κB activation (24). The K215M mutant was an oncogenic mutant from germinal center type (GCB)-DLBCL, which activates downstream NF-κB at the same level as WT *CARD11* (19). Therefore, those two mutations are not LOF mutants.

We found that CARD11 mutations significantly changed Treg development in the thymus. The E134G mutant abolished the Treg generation in the thymus and left almost no CD25^+^ FoxP3^+^ population in the CD4^+^ SP population (**Figure 1C**). On the contrary, the Treg in the thymus of K215M mutant mice was significantly increased, and reached about twice the quantity of Tregs in WT mice (**Figure 1D**). The tTreg development interrupted by the CARD11 mutants also changed the mature Treg population in the secondary lymphoid tissue. The mature splenic Tregs in E134G mutant mice were dramatically reduced to almost none, compared to around 10% of total CD4^+^ T cells in WT spleens (**Figure 1E**). By contrast, the splenic Treg percentage in K215M mutant mice was increased to three times as many as that in WT spleens (**Figure 1F**). To better mimic the genetic conditions in patients whose CARD11 mutations occurred in one allele, we further evaluated the tTreg development in heterozygous *Card11* mutant mice (**Figures 1G–J**; **Supplementary Figures 1D-G**). The single allele E134G mutant still reduced the Treg populations in the thymus and spleen compared to WT mice, but the reduction is milder than that in the homozygous mutant mice (**Figures 1G, H**). The same pattern was observed in K215M heterozygous mutant mice, with mildly increased Treg in the thymus and spleen, compared to WT and homozygous mutant mice (**Figures 1I, J**). The same patterns of Treg absolute counts in the spleen and thymus of *Card11* mutant and KO mice were observed (**Supplementary Figures 1H–M**). Finally, we compared the suppressive function of Tregs carrying distinct *Card11* mutants. Since there are very few Tregs in E134G homozygous mutant mice, we used the Tregs from E134G heterozygous mutant mice in this *in vitro* suppression assay. Tregs purified from WT, heterozygous E134G, and homozygous K215M mutant mice had the same suppressive function on the proliferation of conventional WT CD4^+^ T cells (**Figure 1K**).

Our results demonstrated that the GOF CARD11 mutant, E134G, represents an overactivated NF-κB but still disrupts Treg generation in the thymus. Furthermore, another rare oncogenic mutant, K215M, inducing standard NF-κB activation, increases the Treg population. After comparative analyses of all those *Card11* transgenic mouse models, we concluded that CARD11 regulates Treg development in an NF-κB independent manner.

### CARD11 mutations have distinct influences on CD4^+^ T cell differentiation

**Figure 2:**
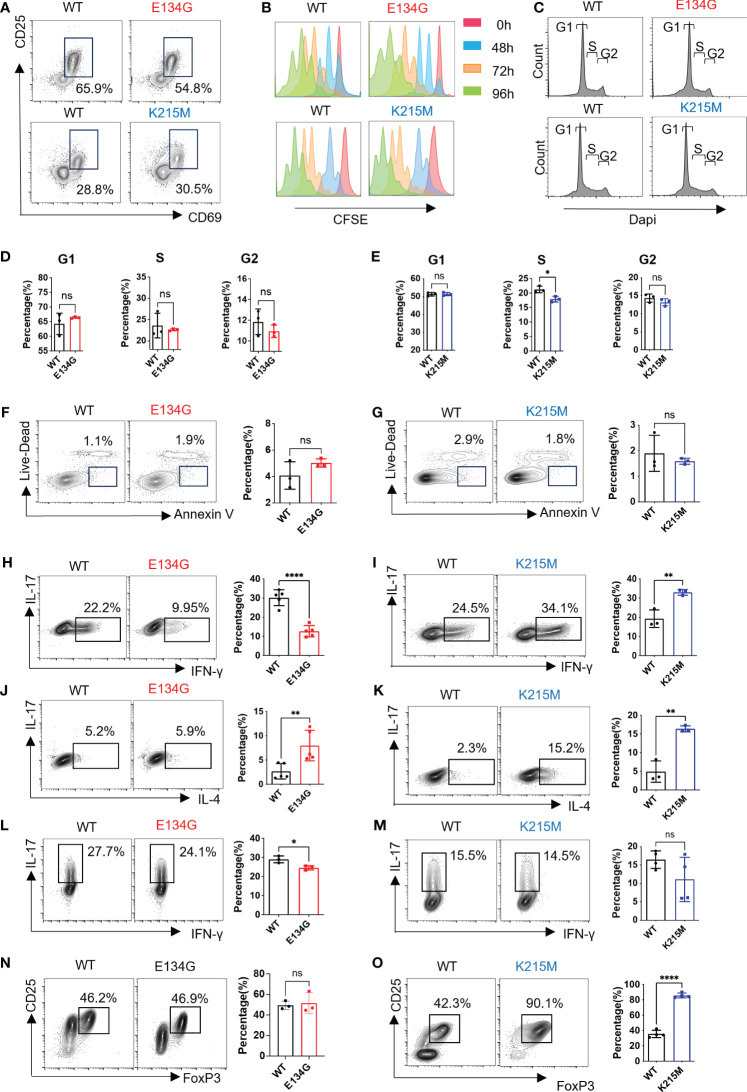
CARD11 mutations have distinct influences on CD4+ T cell differentiation. **(A)** Naïve CD4^+^ T cells were stimulated by anti-CD3 and anti-CD28 (2μg/mL) for 12 hours. CD25 and CD69 expression levels were evaluated by flow cytometry. **(B)** Naïve CD4^+^ T cells in WT and *Card11* mutant mice were stimulated by anti-CD3 and anti-CD28 (2μg/mL) for 0 (red)/48 (blue)/72 (orange)/96 (green) hours after CFDA-SE staining. The CFSE fluorescence intensity was analyzed by flow cytometry after stimulation. **(C)** Naïve CD4^+^ T cells were stained with Dapi, and cell cycle analysis was measured by flow cytometry. **(D, E)** The quantifications of the G1, S, and G2 percentages in WT and E134G mice, as well as, **(E)** WT and K215M mice (n=3). **(F)** Naïve CD4^+^ T cells were stimulated by anti-CD3 and anti-CD28 (2μg/mL) for 48 hours. Apoptotic cell populations (Live-Dead^-^ Annexin^+^) from WT, E134G, and **(G)** K215M mutant mice are shown (left). The quantifications of apoptotic cells in WT, E134G, and K215M mutant mice were shown in the right panel (n=3). **(H, I)** Naïve CD4^+^ T cells were activated and differentiated in the presence of anti-CD3, anti-CD28, and cytokines for 5 days. IFN-γ and IL-17 expression in WT and E134G CD4^+^ T cells, as well as **(I)** WT and K215M CD4^+^ T cells, were analyzed by flow cytometry (left). The quantifications of IFN-γ^+^ Th1 cells (right) (n=3). **(J, K)** IL-4 and IL-17 expression in WT and E134G CD4^+^ T cells, as well as **(K)** WT and K215M CD4^+^ T cells, were analyzed by flow cytometry (left). The quantifications of IL-4^+^ Th2 cells (right) (n=3). **(L, M)** IL-17 and IFN-γ expression in WT and E134G CD4^+^ T cells, as well as **(M)** WT and K215M CD4^+^ T cells, were analyzed by flow cytometry (left). The quantifications of IL-17^+^ Th17 cells (right) (n=3). **(N, O)** CD25 and FoxP3 expression in WT and E134G CD4^+^ T cells, as well as **(O)** WT and K215M CD4^+^ T cells, were analyzed by flow cytometry (left) (n=3). The quantifications of CD25^+^ FoxP3^+^ iTreg cells (right). Data represent the mean ± SEM of n>3 biological replicates. *P<0.05, **P<0.01, ****P<0.0001; P values were determined using unpaired two-tailed Student’s *t-*tests.

Given the crucial role of CARD11 in TCR signal transduction, we next investigate whether the CARD11 mutation will affect CD4^+^ T cell early activation or differentiation. We purified naïve CD4^+^ T cells followed by anti-CD3/CD28 agonist antibody stimulations *in vitro*. After 12 hours of antibody incubation, the WT and CARD11 mutant CD4^+^ T cells were successfully activated. E134G and K215M mutant cells had the same level of CD25 and CD69 upregulation compared to WT cells (**Figure 2A**). Next, we investigated the proliferation of CD4^+^ T cells with various genetic backgrounds. WT CD4^+^ T cells show apparent cell division after 48 hours, and the same level of proliferation was observed in E134G and K215M mutant CD4^+^ T cells (**Figure 2B**). A cell cycle assay by staining proliferative cells with DNA dye, Dapi, suggested that E134G mutant CD4^+^ T cells have equal quantities of cells at the G1, S, and G2 phases compared to WT cells. K215M mutant CD4^+^ T cells also had equivalent populations at the G1 and G2 phases but showed fewer cells at the S phase than WT cells (**Figures 2C–E**). We compared the cell viability by staining activated CD4^+^ T cells with Annexin-V and Cell-Live dye. Both CARD11 mutants showed no impact on cell death compared to WT CARD11 (**Figures 2F, G**).

After activation, CD4^+^ T cells differentiated into different helper T cells under distinct immune microenvironments. We evaluated the differentiation potential of WT and CARD11 mutant CD4^+^ T cells under distinctively skewed conditions toward Th1, Th2, Th17, and iTreg, respectively. E134G mutant cells show defective Th1 differentiation (**Figure 2H**). K215M mutant cells have enhanced Th1 differentiation potential (**Figure 2I**). For the Th2 cells, both the E134G and K215M mutants increased their differentiation, and the K215M mutant had the best Th2-induction potential (**Figures 2J, K**). The E134G mutant also downregulated Th17 differentiation, but the level was minimal (**Figure 2L**). The K215M mutant does not affect Th17 cell differentiation compared to the WT control (**Figure 2M**). Finally, iTreg differentiation did not change in E134G mutant CD4^+^ T cells, but K215M mutant cells show reinforced iTreg differentiation (**Figures 2N, O**).

In summary, CARD11 mutants have no impact on CD4^+^ T cell activation, proliferation, or apoptosis but partially change CD4^+^ T helper cell differentiation potential.

### CARD11 regulates tTreg development through the AKT/FOXO1 pathway

**Figure 3:**
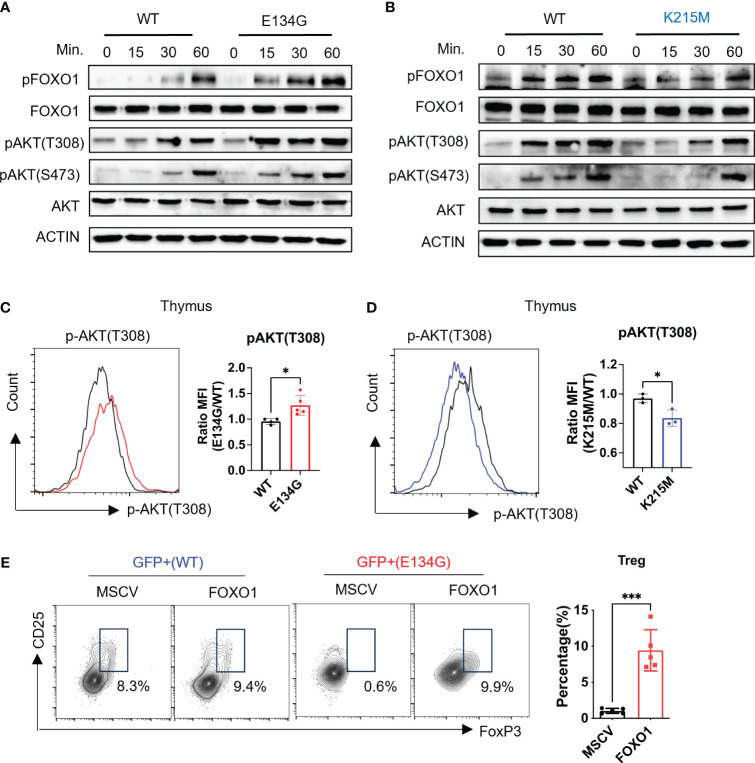
CARD11 regulates tTreg development through the AKT/FOXO1 pathway. **(A, B)** Naïve CD4^+^ T cells from WT and E134G mice and **(B)** WT and K215M mice were stimulated with anti-CD3 and anti-CD28 (2μg/mL) at different time points. The phosphorylations of AKT (T308), AKT (S473), and FOXO1 were measured by western blot. **(C)** CD4^+^ SP Thymocytes of WT and E134G mice, as well as **(D)** WT and K215M mice, were intracellularly stained for p-AKT (T308) and analyzed by flow cytometry (left). The quantifications of pAKT (T308) MFI in WT and *Card11* mutant mice (right) (n=3). **(E)** WT and E134G BM hematopoietic stem cells were transduced with empty retrovirus or FOXO1 overexpression retrovirus, respectively, and then applied to BM chimeras. Six weeks later, GFP^+^ CD4^+^ CD25^+^ FoxP3^+^ Treg cells in the spleens of recipient mice were analyzed by flow cytometry (left). The quantifications of Treg cells are shown in the right panel. (n=5). Data represent the mean ± SEM of n>3 biological replicates. *P<0.05, ***P<0.001; P values were determined using unpaired two-tailed Student’s *t-*tests.

Previous studies demonstrated that CARD11 mutations impact B cell development and differentiation through the AKT/FOXO1 signal axis rather than the NF-κB pathway (25). Therefore, we wonder whether the same regulatory mechanism impacts T cells, especially tTreg development. We purified naïve CD4^+^ T cells from WT and *Card11* mutant mice, followed by *in vitro* stimulation with anti-CD3/CD28 antibodies. Then, we evaluated the downstream TCR signal transduction by measuring the phosphorylation of critical signal proteins at indicated time points. Phosphorylated FOXO1 was detected in WT CD4^+^ T cells after 15 minutes of TCR stimulation. The E134G mutant cells showed increased AKT activation, reflected by stronger phosphorylation at T308 and S473 amino acids. Reinforced AKT activation further exacerbates FOXO1 phosphorylation, which will destabilize FOXO1 protein and interrupt downstream target gene expression (**Figure 3A**). By contrast, the K215M mutant represented an opposite influence on signal transduction, in which phosphorylation of AKT and FOXO1 after TCR stimulation was alleviated (**Figure 3B**).

To confirm that CARD11 mutants changed the AKT/FOXO1 signal pathway *in vivo*, we collected the CD4^+^ SP T cells from the thymus and directly measured the AKT phosphorylation by flow cytometry. We found CD4^+^ SP thymocytes in E134G mutant mice showed elevated phosphorylation levels of AKT (**Figure 3C**). On the contrary, the K215M mutant CD4^+^ SP thymocytes had reduced AKT and FOXO1 phosphorylation (**Figure 3D**). These data demonstrate that the E134G and K215M mutants have the opposite impact on the AKT signal axis in CD4^+^ T cells.

Finally, we tested whether the aberrant AKT signal pathway in E134G mutant CD4^+^ T cells was responsible for the defective tTreg development. As the critical downstream target of the activated AKT signal pathway, FOXO1 is phosphorylated and degraded by the proteasome (26). We applied bone marrow (BM) chimeric mouse models to overexpress exogenous FOXO1 in E134G-mutated hematopoietic stem cells by retroviral transduction. After 8 weeks of reconstitution, we collected the thymus from chimeric mice and evaluated the tTreg development. Extra FOXO1 did rescue the tTreg development defects in E134G mutant cells (**Figure 3E**). These findings support the idea that CARD11 mutants regulate the development of tTreg independently of NF-κB.

### CARD11 mutation patients have Treg defects and autoimmune phenotypes *in vivo*

Although transgenic mice with BENTA-associated CARD11 mutations show tTreg development defects, whether the BENTA patients represent the same phenotype is unclear. Therefore, we set up a small PID cohort to recruit patients carrying CARD11 mutations and verify the functional effects of those mutations on the Treg lineage.

**Figure 4:**
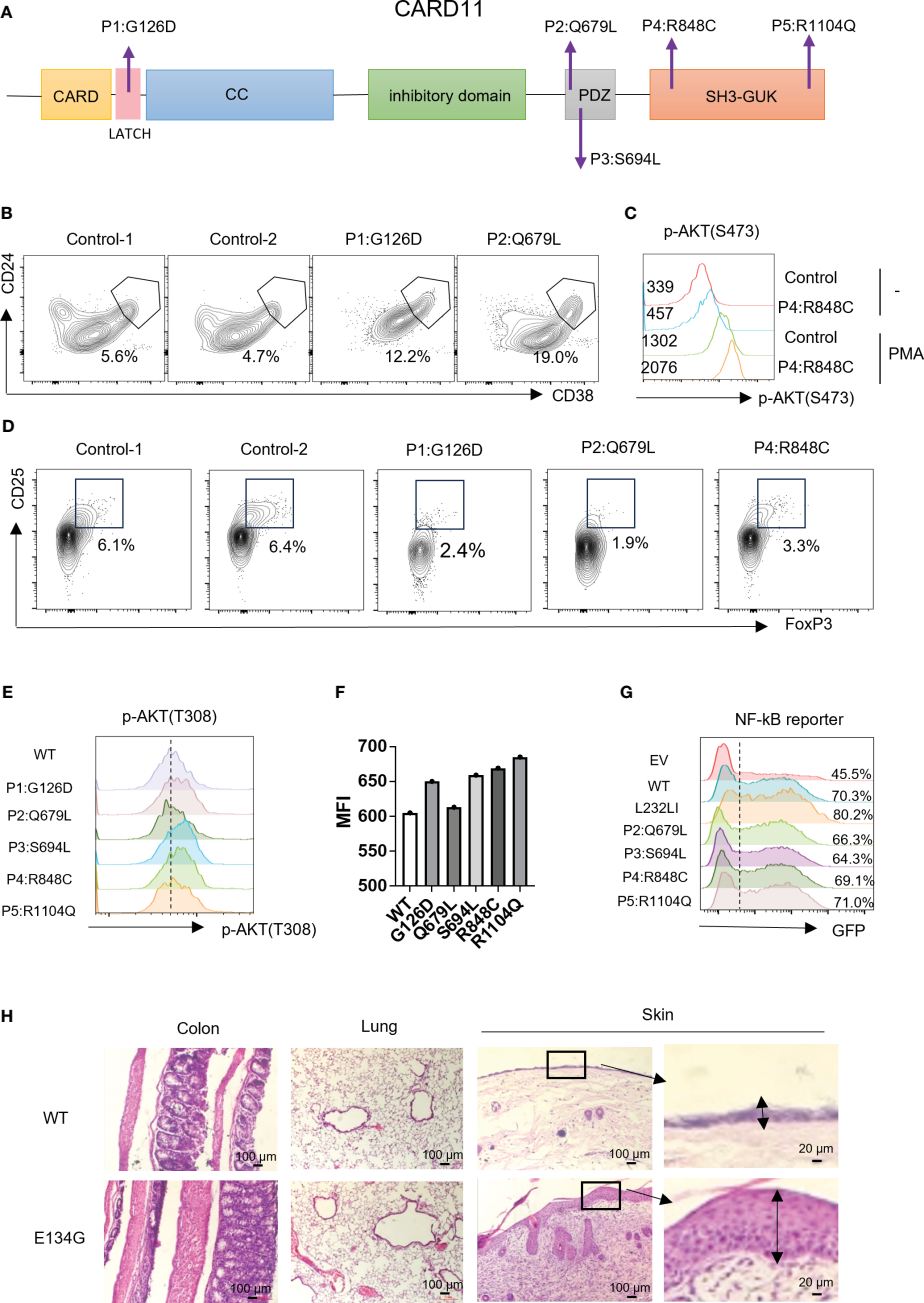
CARD11 mutation patients have Treg defects and autoimmune phenotypes *in vivo*. **(A)** Schematic of CARD11 protein domains showing the location of mutations identified in patients. **(B)** CD24 and CD38 expression on B cells in PBMCs of patients and healthy controls. The black frame indicates the population of transitional B cells. **(C)** The p-AKT (S473) was evaluated on CD4^+^ T cells in PBMCs of healthy control and P4 (R848C) before and after PMA stimulation. The number indicates the MFI of each group. **(D)** CD25 and FoxP3 expression on CD4^+^ T cells in PBMCs of patients and healthy controls. The black frame indicates the population of Treg cells. **(E)** Plasmids expressing the *CARD11* mutants from patients were transfected into human T cells, and the p-AKT (T308) was evaluated by flow cytometry. **(F)** The quantification of the MFI of p-AKT (T308). **(G)** The *CARD11* mutants from these patients were transfected into HEK-293T cells with NF-κB reporter plasmids. The reporter GFP expression was evaluated by flow cytometry. **(H)** Hematoxylin and eosin (HE) staining images of the colon, lung, and skin. The dark box indicates lymphocyte infiltration, and the data represent the mean ± SEM of n>3 biological replicates.

According to the clinical and lab investigations, we finally confirmed 5 PID patients whose symptoms and genetic modifications were similar to those previously reported in CARD11 mutant patients (**Figure 4A**; **Table 1**). P1 in this cohort had a CARD11 heterozygous mutation at G126D located in the Latch domain (27), where most BENTA-associated CARD11 mutants were initially discovered. The G126D mutant was reported in one BENTA patient published before (28) and induced an autonomously activated NF-κB pathway (27). Mutations in the other four patients are at the C-terminal of the CARD11 protein. The P2 (Q679L) and P3 (S694L) mutations are in the PDZ domain. P4 (R848C) and P5 (R1104Q) mutants are in the SH3-GUK domain. Although all five patients had an elevated percentage of transitional B cells, a type of immature B cells (**Figure 4B**; **Table 2**), only P1 represents the B cell lymphocytosis phenotype that is frequently observed in BENTA patients (**Table 2**). The other four patients have normal quantities of B cells in their peripheral blood (**Table 2**). Patients have an average naïve T cell population in their PBMC, indicating normal conventional T cell development. All the other immune cell types are in the normal range (**Table 2**). After PMA treatment to mimic the antigen stimulations, CD4^+^ T cells from one healthy control and patient P4 showed upregulated AKT S473 phosphorylation, and cells from P4 showed higher AKT activation (**Figure 4C**). It recapitulates the hyper-activated AKT signaling in CD4^+^ T cells from E134G mutant mice. Then, we collected the peripheral blood from 3 patients (P1, P2, and P4) and evaluated their Treg population by flow cytometry. Healthy control samples always showed that 5-10% of the total CD4^+^ T cells are FoxP3^+^ CD25^+^ Tregs. However, all 3 patients have less Tregs in their peripheral blood (**Figure 4D**; **Table 2**). These results suggest that patients with pathogenic *CARD11* mutations also have defective Treg populations, as in mouse models. We also evaluated AKT T308 phosphorylation and NF-κB activation by transfecting cells with patient-derived *CARD11* mutant plasmids. In contrast to WT, overactivated phospho-AKT (T308) was observed in human T cells transfected with *CARD11* mutant plasmids (**Figures 4E, F**). However, there was no significant difference in CARD11 mutants (P2-P5) induced NF-κB activation in a GFP-based reporter assay compared to WT CARD11 (**Figure 4G**).

**Table 1:** Clinical characteristics of patients at diagnosis.

	Gender	Onset age	Mutation	Recurrent fever	RPTI	Atopy	Arthritis	Lymphadenopathy	Hepatomegaly	Splenomegaly	Other presentations
P1	Male	5m	c.377G>A p.G126D	Yes	Yes	No	No	Yes (cervix, inguen and enterocoelia)	Yes	Yes	Hemophagocytic syndrome
P2	Male	10m	c. 2036A>T p.Q679L	Yes	Yes	Yes(allergic dermatitis, allergic rhinitis)	No	No	No	No	Patent ductus arteriosus
P3	Male	21m	c.2081C>T p.S694L	Yes	Yes	Yes (asthma)	No	No	No	No	No
P4	Male	12m	c. 2542C>T p.R848C	Yes	Yes	Yes(rash and allergic rhinitis)	Yes	Yes(cervix)	No	No	No
P5	Male	8m	c.3311G>A p.R1104Q	Yes	Yes	No	No	Yes (cervix and inguen)	No	No	No

RRTI, recurrent respiratory tract infections.

**Table 2:** Immunological characteristics.

Lymphocyte subpopoulation	P1	P2	P3	P4	P5	Reference values
T cells %	37.08↓	33.74	72.41	76.94	65.36	60.05-74.08
T cell counts (cells/uL)	1711.9	927.1↓	1775	2503.7	1923.2	1424-2664
Th cells (CD3^+^CD4^+^)%	21.18↓	15.65↓	38.03	32.71	38.99	24.00-38.72
Th cell counts (cells/uL)	977.95	430.07↓	932.22	1064.8	1147.29	686-1358
Naive Th cells (CD4^+^CD45RA^+^CD27^+^)	75	81↑	71.8	65.4	79.3↑	45.56-75.28
Central memory Th cells (CD4^+^CD45RA^-^CD27^+^)	23.7	18.2↓	22.8	27.3	19.9↓	22.06-46.46
Effector memory Th cells (CD4^+^CD45RA^-^CD27^-^)	1.2↓	0.7↓	5.4	6.0	0.8↓	1.08-8.78
Cytotoxic T cells (CD3^+^CD8^+^)	14.08↓	14.18↓	26.95	39.36↑	22.23	19.68-34.06
Cytotoxic T cell counts (cells/uL)	649.86	389.53↓	660.72	1280.78↑	654.09	518-1125
Naive cytotoxic T cells (CD8^+^CD45RA^+^CD27^+^)	63.8	87.7↑	80.8↑	69.4	87.5↑	41.58-77.90
Central memory cytotoxic T cells (CD8^+^CD45RA^-^CD27^+^)	8.3↓	11.7↓	15.1	12.3	6.68↓	12.08-30.54
Effector memory cytotoxic T cells (CD8^+^CD45RA^-^CD27^-^)	9.3	0.5↓	3.54	3.4	1.8	1.58-13.18
CD4/CD8	1.5	1.1	1.41	0.83↓	1.75	0.87-1.94
TCRαβ+ double negative T cells (CD3^+^CD4^-^CD8^-^)%	5.4	2.63↓	5.85	10.8	7.45	4-55
B-cell (CD19^+^)%	57.71↑	45.93↑	19.21	16	19.52	10.21-20.12
B-cell counts (cells/uL)	2663.95↑	1262.01↑	470.82	520.57	547.4	280-623
Naive B cells (CD19^+^IgD^+^CD27^-^)	97.8↑	95.8↑	94.2↑	67.7	95.4↑	48.36-75.84
Memory B cells (CD19^+^IgD^-^CD27^+^)	0.1↓	0.65↓	1.36↓	8.5	0.75↓	7.76-19.9
Transitional B cells (CD19^+^CD24^+^CD38^+^)	29.8↑	43.1↑	26.9↑	26.6↑	24.8↑	2.58-12.3
Transitional B cell counts (cells/uL)	793.86↑	543.93↑	126.65↑	138.47↑	135.83↑	10-66
Plasmablasts	0.4↓	0.6↓	0.9	4.5	0.6↓	0.9-7.36
NK cell (CD16^+^CD56^+^)%	4.65↓	18.67	6.85↓	6.2↓	13.64	9-22.24
Regulatory T cell(CD4^+^CD25^+^FoxP3^+^)%	2.4↓	1.9↓	NA	3.3↓	NA	5-10
Regulatory T cell Counts	23.47↓	8.17↓	NA	35.14	NA	34.3-135.8
NK cell counts (cells/uL)	214.52↓	512.91	167.93↓	201.62↓	401.48	258-727
Immunoglobulin						
IgG (g/L)	9.8	9.13	4.6↓	8.02	6.36	4.95-12.74
IgA (g/L)	0.14↓	0.99	0.55	0.78	0.35	0.33-1.89
IgM (g/L)	0.95	1.66	0.35↓	0.89	0.21↓	0.65-2.1
IgE (g/L)	21.6	321.1↑	423.7↑	35.2	11	<100

The symbol “ ↓”means that decrease and "↑" means that "increase".

Tregs play an essential role in preventing autoimmune pathology *in vivo*. Since CARD11 mutants in patients cause Treg development defects, we wonder whether autoimmune syndromes are present. P1 with the G126D mutation is a typical BENTA patient. Like other reported BENTA patients, P1 did not show general autoimmunity but had hemophagocytic syndrome, which is also associated with an overactivated immune system (**Table 2**). Patients P2, P3, and P4 represented atopic syndrome as observed in CADINS patients with LOF CARD11 mutations (22, 23). P2 showed itching of the eyes, edema of the eyelids, and rubbed eyes repeatedly exposed to pollen, which developed into atopic dermatitis (recurrent itchy skin of the scalp, limbs, and trunk). P3 had recurrent coughing and sneezing. The symptoms were significantly relieved after treatment with glucocorticoids and H1 receptor antagonists. Repeated sneezing, a runny nose, and thickening of the nasal mucosa characterized P4. The symptoms were relieved after treatment with glucocorticoids and leukotriene antagonists (**Table 2**). We re-checked our mouse models since BENTA patients did not show general autoimmune syndromes. We found that the CARD11 E134G mutant did cause severe inflammatory dermatitis, reflected by severe lymphocyte infiltration and thickened dermal epidermis when those mice reached six months of age. However, no such phenotype was observed in colon and lung tissues (**Figure 4H**). It suggests that GOF CARD11 mutants also cause abnormal dermatological phenotypes, as do LOF CADINS mutants. Those data indicate that the compromised Treg generation may be the reason for atopy. However, only the skin-specific autoimmune responses indicated that the dermal immune microenvironment is the most sensitive to the tTreg decrease.

In conclusion, CARD11 regulates tTreg development through its NF-κB-independent function. These new findings extend our knowledge about the critical role of CARD11 in immune cell lineage determination and functional differentiation.

## Discussion

Scaffold protein CARD11 in T cells transduces the antigen receptor signal to the downstream TFs, initiating T cell activation and differentiation (29). The proximal TCR signal triggers a cascade of kinases to phosphorylate CARD11. It changes its conformation structure from an auto-inhibition state to an active, open state activating NF-κB. Given the crucial role of NF-κB in the immune system, aberrant CARD11 mutations in patients cause many severe immunological disorders (30, 31). Recently, new results indicate that the regulatory function of CARD11 is more complicated than we expected. After investigating distinctive pathogenic CARD11 mutants, data demonstrated that CARD11 modulates other downstream targets besides NF-κB (29, 32, 33). BENTA disease-associated E134G mutant and oncogenic K215M mutant identified from DLBCL have opposite impacts on AKT activations in B cells (25). Another study on the CARD11 L251P mutant implied that CARD11 could regulate the mTOR signal activation (32). These results suggest that CARD11 has diverse regulatory functions in B cells. However, whether these NF-κB-independent functions of CARD11 also impact T cells is unclear.

*Card11-*deficient mouse models have demonstrated that CARD11 is dispensable for conventional T cell development, but the regulatory T cells are dramatically decreased in the thymus of *Card11* KO mice. Without *Card11*, the Treg development defect recapitulates the phenotypes in NF-κB knockout mice, in which tTreg development was also severely impaired (17, 18). It has proposed that CARD11 regulates tTreg development through its NF-κB regulatory functions. Later, some insightful studies demonstrated that distinctive *Card11* mutations influenced Treg development. The CARD11 M365K mutant was initially found in DLBCL. Like many other oncogenic mutants, the M365K mutant induces enhanced NF-κB activation in lymphocytes. The Treg population was significantly increased in the M365K mutant mouse model (34). The R30W CARD11 mutant, found in CADINS patients, is a dominant negative mutant suppressing NF-κB activation. R30W mutant mice had a decreased Treg population (35). The unmodulated mutant mice carrying an L298Q CARD11 mutant, which attenuates the downstream TCR signaling through CARD11, also have a smaller Treg population. Most importantly, R30W and unmodulated mice show elevated IgE and atopic dermatitis (35, 36). The atopic dermatitis phenotype in E134G mutant mice is similar to those in R30W and unmodulated mouse models (35, 36). We have not measured the IgE level in E134G mutant mice. However, Th2 cell differentiation was enhanced in E134G mutant CD4^+^ T cells.

Since the E134G mutant identified in BENTA disease patients induces an autonomously activated NF-κB in T cells, we initially thought of better Treg generation in E134G mutant mice. Unexpectedly, this GOF CARD11 mutation disrupted the tTreg development similarly to the defects in *Card11* deficient mice and R30W, as well as unmodulated mutant mice. Moreover, another oncogenic CARD11 mutant, K215M, inducing a standard NF-κB activation as WT CARD11, significantly increases the tTreg population. These data challenge the view that CARD11 regulates tTreg development through NF-κB. We noticed that the E134G mutant and K215M mutant also gave rise to opposite impacts on AKT signal activation in CD4^+^ T cells. As in B cells, the E134G mutant enhances AKT activation in CD4^+^ T cells after antigen engagement. On the contrary, the K215M mutant alleviates AKT activation. Hence, we proposed a new model in which CARD11 regulates tTreg development through the AKT/FOXO1 pathway. Activated AKT phosphorylates FOXO1 and then promotes FOXO1 degradation. FOXO1 is an indispensable TF required for Treg development and differentiation (26). Previous studies have suggested that the absence of *Foxo1* in mouse CD4^+^ T cells dramatically reduced the tTreg generation (37, 38). Therefore, re-introducing exogenous FOXO1 in E134G mutant progenitor cells rescued the tTreg generation, confirming that an NF-κB-independent regulatory pathway mediated by CARD11 is responsible for tTreg development.

However, the puzzle is still not fully solved. Although tTreg development is severely interrupted in E134G mutant mice, no general autoimmunity was observed in the E134G mutant mice except for dermatitis with skin pathology. Moreover, unlike the mouse model, not all the BENTA patients showed autoimmune syndromes and signs of dermatitis. This discrepancy may be due to the haplosufficiency of CARD11 since we have not found a similar skin abnormality in the heterozygous E134G mutant mice either, which may explain the difference between mouse models and patients. Secondly, it emphasizes the complexity of the human immune system, which constantly faces environmental challenges. By contrast, mouse models under specific pathogen-free conditions may not ideally mimic human diseases. Moreover, as a rare disease, the limited number of PID patients also leads to variations. Nevertheless, further studies focusing on the immune microenvironment in the skin may illuminate the answers.

Our study emphasized the importance of CARD11 in the Treg lineage determination. It’s not the NF-κB but the AKT/FOXO1 signal axis regulated by CARD11 and its pathogenic mutants responsible for Treg development. Our findings provide extra clues to understanding the pathogenesis of BENTA, B-cell lymphoma, and other CARD11-related immunological disorders. The proposed model may help develop diagnostic and therapeutic strategies shortly.

## Data availability statement

The original contributions presented in the study are included in the article/**Supplementary Material**. Further inquiries can be directed to the corresponding authors.

## Ethics statement

The studies involving humans were approved by The Ethics Board of Children’s Hospital, Fudan University. The studies were conducted in accordance with the local legislation and institutional requirements. Written informed consent for participation in this study was provided by the participants’ legal guardians/next of kin. The animal study was approved by Institutional Biomedical Research Ethics Committee of the Shanghai Institute of Nutrition and Health Science, Chinese Academy of Sciences. The study was conducted in accordance with the local legislation and institutional requirements.

## Author contributions

YH: Writing – original draft, Writing – review & editing. LH: Data curation, Resources, Writing – review & editing. WX: Formal analysis, Methodology, Writing – review & editing. TL: Writing – review & editing. QZ: Formal analysis, Investigation, Software, Writing – review & editing. WL: Funding acquisition, Supervision, Writing – review & editing. JS: Funding acquisition, Supervision, Writing – review & editing. YW: Funding acquisition, Project administration, Resources, Supervision, Writing – review & editing.

## Supplementary material

Supplementary Figure 1 Phenotype analysis of WT, heterozygous, and homozygous mutant mice. **(A)** Schematic of CARD11 showing the location of E134G and K215M mutations on the distinct protein domains. The color of the mutations indicates the different diseases. **(B)** The thymus and **(C)** spleens morphology of WT, E134G, and K215M mutant mice. **(D)** CD25 and FoxP3 expression on CD4^+^ T cells in the thymus and **(E)** spleens of WT, heterozygous, and homozygous E134G mutant mice. **(F)** CD25 and FoxP3 expression on CD4^+^ T cells in the thymus and **(G)** spleens of WT, heterozygous, and homozygous K215M mutant mice. Data represent mean ± SEM of n>3 biological replicates. **(H)** The absolute counts of tTreg in the spleen and **(I)** thymus of WT and E134G mutant mice were calculated after collecting a total amount of 300,000 cells. **(J)** The absolute counts of tTreg in the spleen and **(K)** thymus of WT and K215M mutant mice were calculated as in **(H, I)**. **(L)** A total amount of 220,000 cells were collected. The absolute counts of tTreg in the spleen and **(M)** thymus of WT and CARD11 KO mice were calculated. Data represent the mean ± SEM of n>3 biological replicates.
