# Supplementary material for: CARD11 regulates the thymic Treg development in an NF-κB-independent manner
Source: Front Immunol. 2024 Apr 8;15:1364957. doi: 10.3389/fimmu.2024.1364957 (PMC11033321; doi:10.3389/fimmu.2024.1364957)
Supplement: Supplementary Figure 1 — Phenotype analysis of WT, heterozygous, and homozygous mutant mice. (A) Schematic of CARD11 showing the location of E134G and K215M mutations on the distinct protein domains. The color of the mutations indicates the different diseases. (B) The thymus and (C) spleens morphology of WT, E134G, and K215M mutant mice. (D) CD25 and FoxP3 expression on CD4+ T cells in the thymus and (E) spleens of WT, heterozygous, and homozygous E134G mutant mice. (F) CD25 and FoxP3 expression on CD4+ T cells in the thymus and (G) spleens of WT, heterozygous, and homozygous K215M mutant mice. Data represent mean ± SEM of n>3 biological replicates. (H) The absolute counts of tTreg in the spleen and (I) thymus of WT and E134G mutant mice were calculated after collecting a total amount of 300,000 cells. (J) The absolute counts of tTreg in the spleen and (K) thymus of WT and K215M mutant mice were calculated as in (H, I). (L) A total amount of 220,000 cells were collected. The absolute counts of tTreg in the spleen and (M) thymus of WT and CARD11 KO mice were calculated. Data represent the mean ± SEM of n>3 biological replicates. [file DataSheet_1.docx]

Supplementary Material


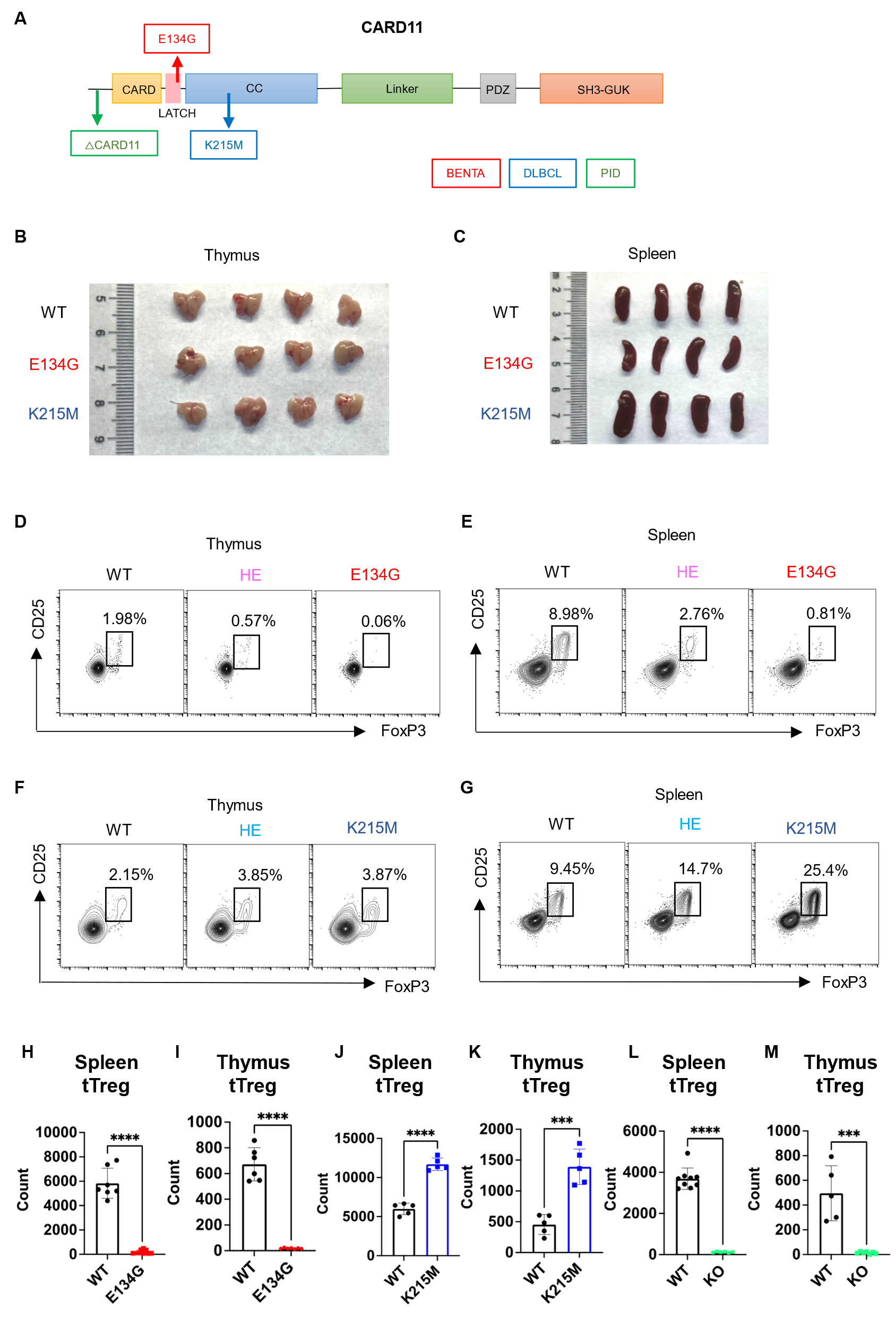


**Figure S1 Phenotype analysis of WT, heterozygous, and homozygous mutant mice.** (**A**) Schematic of CARD11 showing the location of E134G and K215M mutations on the distinct protein domains. The color of the mutations indicates the different diseases. (**B**) The thymus and **(C)** spleens morphology of WT, E134G, and K215M mutant mice. (**D**) CD25 and FoxP3 expression on CD4^+^ T cells in the thymus and **(E)** spleens of WT, heterozygous, and homozygous E134G mutant mice. (**F**) CD25 and FoxP3 expression on CD4^+^ T cells in the thymus and (**G**) spleens of WT, heterozygous, and homozygous K215M mutant mice. Data represent mean ± SEM of n>3 biological replicates. (**H**) The absolute counts of tTreg in the spleen and (**I**) thymus of WT and E134G mutant mice were calculated after collecting a total amount of 300,000 cells. (**J**) The absolute counts of tTreg in the spleen and (**K**) thymus of WT and K215M mutant mice were calculated as in **(H)** and **(I)**. (**L**) A total amount of 220,000 cells were collected. The absolute counts of tTreg in the spleen and (**M**) thymus of WT and CARD11 KO mice were calculated. Data represent the mean ± SEM of n>3 biological replicates.
